# Additive impulsivity and emotion dysregulation in adolescents with comorbid bipolar and substance use disorder: a cross-sectional factorial study

**DOI:** 10.3389/fpsyt.2026.1825855

**Published:** 2026-06-05

**Authors:** Fevzi T. Ocakoglu, Yiğit Özaydın, Naz Arslan, Birsen Şentürk Pilan, Tezan Bildik, Eyup S. Ercan

**Affiliations:** Department of Child and Adolescent Psychiatry, Faculty of Medicine, Ege University, İzmir, Türkiye

**Keywords:** adolescence, bipolar disorder, comorbidity, emotion regulation, factorial design, impulsivity, substance use disorder, transdiagnostic

## Abstract

**Introduction:**

Comorbid bipolar disorder (BD) and substance use disorder (SUD) in adolescence is associated with poor clinical outcomes, yet the independent and interactive contributions of impulsivity and emotion dysregulation remain poorly understood.

**Methods:**

This cross-sectional study employed a 2 × 2 factorial design to examine impulsivity, measured with the Barratt Impulsiveness Scale-11, and emotion regulation difficulties, measured with the Difficulties in Emotion Regulation Scale, across four groups of adolescents (N = 128; aged 12–18 years): BD+SUD (n = 32), BD-only (n = 32), SUD-only (n = 32), and healthy controls (n = 32). All clinical participants were assessed during euthymia. Factorial analyses of covariance controlled for age, sex, residence, family structure, and income.

**Results:**

Significant BD × SUD interactions were found for emotion regulation, F(1,120) = 35.89, p < .001, ηp^2^ = .230, and impulsivity, F(1,120) = 9.51, p = .002, ηp^2^ = .073. The BD+SUD group showed the highest scores on both measures, exceeding the SUD-only group by 38.90 points on emotion dysregulation and 26.72 points on impulsivity. In the substance-using subsample (n = 64), impulsivity was the strongest predictor of substance use severity (B = 0.61, p < .001; R^2^ = .48). The BD+SUD group also displayed earlier illness onset, mixed-feature predominance, greater polydrug use, and exclusive high-lethality suicide attempts. Low income was the strongest exploratory predictor of clinical group membership.

**Discussion:**

These findings support an additive comorbidity model in which BD and SUD jointly amplify impulsivity and emotion dysregulation, and they highlight the need for integrated, impulsivity-focused interventions in adolescents with dual diagnoses.

## Introduction

Bipolar disorder (BD) is a chronic, recurrent mood disorder characterized by episodes of mania, hypomania, and depression, accompanied by marked impairments in emotional, cognitive, and social functioning (1). Substance use disorder (SUD), defined as the maladaptive use of or dependence on alcohol and/or illicit substances, is among the most prevalent and clinically significant comorbidities associated with BD across both adult and adolescent populations. Large-scale epidemiological investigations consistently demonstrate that individuals with BD exhibit substantially elevated rates of SUD relative to the general population, with lifetime co-occurrence estimates ranging from 30% to 60% (2, 3). In adolescents, this comorbidity is particularly concerning: BD+SUD presentations are associated with earlier illness onset, greater episode frequency, poorer treatment response, and elevated rates of suicidality compared to either disorder alone (4, 5).

Adolescence represents a neurodevelopmentally sensitive period characterized by accelerated structural and functional maturation of prefrontal–limbic circuitry. This developmental imbalance—marked by relatively heightened limbic reactivity alongside still-maturing prefrontal regulatory systems—has been implicated in normative increases in impulsivity, emotional reactivity, and risk-taking behavior (6, 7). When early-onset BD emerges during this period, it frequently presents with pronounced emotional lability and impaired inhibitory control, features that may further amplify vulnerability to substance initiation and escalation. Consistent with this view, adolescents with comorbid BD and SUD display more severe clinical profiles than those with either diagnosis alone, including greater academic impairment, higher rates of psychiatric hospitalization, and increased suicidal behavior (4, 8).

Impulsivity and emotion regulation difficulties are increasingly conceptualized as transdiagnostic dimensions that cut across traditional diagnostic boundaries (9, 10). Both constructs reflect disruptions in top-down prefrontal regulatory control over limbic reactivity and are implicated in the development and maintenance of both mood and substance-related disorders (11, 12). In adult samples, factorial designs comparing BD-only, SUD-only, BD+SUD, and healthy control groups have yielded evidence of distinct clinical profiles, with additive or interactive effects across diagnostic dimensions on measures of impulsivity and affect dysregulation (13–15). Convergent evidence from non-factorial designs further indicates that impulsivity and emotion dysregulation, although correlated, capture partially distinct regulatory vulnerabilities: dimensional studies in non-clinical adolescents have documented robust associations between impulsivity facets and emotion regulation difficulties (16), while clinical investigations in adult SUD samples have established emotion dysregulation as a salient feature of substance-related pathology (17). Whether these two dimensions exert independent, additive, or interactive effects when BD and SUD co-occur—particularly in adolescence—remains an open question. Although adolescent studies have separately examined impulsivity and emotion regulation in BD or in SUD, factorial designs that simultaneously cross BD and SUD status to disentangle their independent and interactive contributions in adolescent samples are, to our knowledge, lacking. This represents a critical gap given the neurodevelopmental sensitivity of the prefrontal–limbic systems implicated in both constructs and the heightened clinical severity associated with adolescent-onset BD+SUD comorbidity.

Affective instability and impulsivity have been increasingly recognized not merely as symptom-level features of individual disorders but as transdiagnostic dimensional constructs that cut across categorical diagnostic boundaries (9–11). Affective instability—characterized by frequent, disproportionate, and poorly regulated shifts in emotional state—has been documented across bipolar disorder, borderline personality disorder, and substance-related conditions, suggesting a shared underlying disruption in prefrontal–limbic regulatory circuitry (18, 19). Similarly, impulsivity, as a multidimensional construct encompassing attentional, motor, and non-planning components, has been consistently implicated in the etiology and clinical course of mood and addictive disorders alike (20, 21). The convergence of these two transdiagnostic dimensions—affective instability and impulsivity—in the context of comorbid BD and SUD may therefore produce a compound regulatory deficit that exceeds the burden conferred by either disorder individually. Examining these dimensions jointly within a factorial framework offers a methodological opportunity to disentangle their independent and interactive contributions, an approach that has been underutilized in adolescent populations (12).

The present study employed a cross-sectional 2×2 factorial design to examine impulsivity and emotion regulation across four groups of adolescents: BD+SUD, BD-only, SUD-only, and healthy controls (HC). The design does not permit causal inference, and findings should not be interpreted as establishing directional or mechanistic relationships between variables. Academic functioning and sociodemographic characteristics were evaluated as secondary descriptive parameters to contextualize group differences. Given the use of a clinical convenience sample from a tertiary psychiatric service, findings are not epidemiologically representative and should not be generalized to population-level prevalence estimates of BD-SUD comorbidity.

Based on the existing literature, we formulated the following hypotheses: Hypothesis 1: Adolescents with comorbid BD+SUD will exhibit significantly higher levels of impulsivity compared to all other groups (BD-only, SUD-only, and HC), consistent with an additive or synergistic effect of co-occurring diagnoses on impulsive behavioral control. Hypothesis 2: Both BD-only and SUD-only groups will demonstrate significantly higher impulsivity and emotion regulation difficulties relative to healthy controls, reflecting the established clinical burden associated with each disorder independently. Hypothesis 3 (exploratory): Observed group differences in impulsivity and emotion regulation may be associated with selected clinical and sociodemographic variables. Given the number of comparisons involved, all secondary and exploratory analyses should be interpreted with appropriate caution.

## Materials and methods

### Study design and participants

This study employed a cross-sectional, observational design using a clinical convenience sample. Due to its cross-sectional nature, causal inferences cannot be drawn and findings are interpreted as associations between diagnostic status and clinical characteristics. Participants were adolescents aged 12–18 years recruited from outpatient psychiatry and addiction medicine clinics. A total of 128 individuals were allocated into four equal groups: bipolar disorder with substance use disorder (BD+SUD, n = 32), bipolar disorder without SUD (BD-only, n = 32), SUD without BD (SUD-only, n = 32), and healthy controls (n = 32). Group comparisons were structured within a 2×2 factorial framework to evaluate the independent and interactive effects of BD (present/absent) and SUD (present/absent) on impulsivity and emotion regulation.

Participants were included if they met DSM-5 criteria for BD and/or SUD according to structured clinical interview, were between 12 and 18 years of age, and provided written informed consent from both themselves and their legal guardians. All clinical participants were required to be in a euthymic state at assessment, defined as a Young Mania Rating Scale (YMRS) score ≤ 7 and a Beck Depression Inventory (BDI) score ≤ 10, sustained for at least four consecutive weeks prior to evaluation.

The four-week euthymic period was established through triangulated assessment: structured clinical interview with the participant and legal guardian, retrospective self- and caregiver report of the absence of affective episodes or significant mood fluctuations during the preceding four weeks, and systematic review of outpatient medical records including prior YMRS and BDI scores where documented. Cases for which euthymia stability could not be reliably confirmed across all three sources were excluded.

Although group-level YMRS means may slightly exceed 7 due to distributional variability, all individual participants met the euthymia threshold at assessment. Exclusion criteria included schizophrenia or other primary psychotic disorders (psychotic features occurring exclusively during BD episodes were not exclusionary), neurological or severe medical conditions, intellectual disability (IQ < 70), and language or cognitive impairments interfering with valid assessment.

Diagnoses were established according to DSM-5 criteria (1) using the Schedule for Affective Disorders and Schizophrenia for School-Age Children—Present and Lifetime Version (K-SADS-PL-DSM-5; 22), administered by trained child and adolescent psychiatrists. Comorbid psychiatric disorders were systematically assessed; primary psychotic disorders were exclusionary, whereas other comorbidities (e.g., ADHD, anxiety disorders) were recorded but did not determine group allocation. The distribution of comorbid psychiatric diagnoses across groups is reported in **Table 1** as a supplementary descriptive parameter to contextualize potential confounding.

**Table 1:** Sociodemographic, clinical, and pharmacological characteristics across groups.

Variable	SUD (n=32)	BD+SUD (n=32)	BD (n=32)	HC (n=32)	Test	p / ES
Demographics						
Age (years), M ± SD	15.8 ± 1.3	14.9 ± 1.6	15.3 ± 1.7	15.8 ± 1.1	F = 2.58	.056 η² = .06
Sex (Male), n (%)	19 (59.4)	16 (50.0)	25 (78.1)	20 (62.5)	χ² = 5.60	.133 V = .21
Residence (Urban), n (%)	24 (75.0)	21 (65.6)	32 (100)	32 (100)	χ² = 35.07	< .001* V = .52
Nuclear Family, n (%)	19 (59.4)	18 (56.3)	18 (56.3)	28 (87.5)	χ² = 19.12	.004* V = .39
Maternal Education, M ± SD	3.0 ± 1.0	2.8 ± 1.1	2.8 ± 0.9	4.2 ± 1.2	F = 12.35	< .001* η² = .23
Academic / functional						
School Dropout, n (%)	20 (62.5)	24 (75.0)	0 (0.0)	0 (0.0)	χ² = 68.15	< .001* V = .73
Grade Retention, n (%)	15 (46.9)	12 (37.5)	0 (0.0)	0 (0.0)	χ² = 35.06	< .001* V = .52
Currently Employed, n (%)	9 (28.1)	12 (37.5)	0 (0.0)	0 (0.0)	χ² = 26.14	< .001* V = .45
Familial clinical history						
Paternal Psychiatric Dx, n (%)	4 (12.5)	10 (31.3)	0 (0.0)	1 (3.1)	χ² = 28.83	.001* V = .47
Maternal Psychiatric Dx, n (%)	3 (9.3)	9 (28.1)	7 (21.8)	2 (6.2)	χ² = 24.72	< .001* V = .44
Paternal Substance Use, n (%)	4 (12.5)	8 (25.0)	0 (0.0)	1 (3.1)	χ² = 13.27	.004* V = .32
Maternal Alcohol Use, n (%)	1 (3.1)	8 (25.0)	2 (6.2)	1 (3.1)	χ² = 13.30	.038* V = .32
Bipolar clinical features						
BD Onset Age, M ± SD	—	13.6 ± 1.6	14.5 ± 1.7	—	t = −2.48	.015* d = 0.62
Psychotic Features, n (%)	—	7 (21.9)	1 (3.1)	—	χ² = 5.17	.023* V = .28
Suicide Attempt, n (%)	—	16 (50.0)	10 (31.2)	—	χ² = 2.45	.120 V = .20
High-Lethality Attempt, n (%)	—	5 (15.6)	0 (0.0)	—	Fisher	.015*
Hospitalization (Ever), n (%)	—	6 (18.8)	7 (21.9)	—	χ² = 0.10	.754
Pharmacological treatment (BD groups only)						
Mood Stabilizer, n (%)	—	27 (84.4)	26 (81.3)	—	χ² = 0.10	.540
Antipsychotic, n (%)	—	30 (93.8)	29 (90.6)	—	χ² = 0.22	.834
Combination AP Use, n (%)	—	18 (56.3)	17 (53.1)	—	χ² = 0.06	.812

SUD, Substance Use Disorder; BD, Bipolar Disorder; HC, Healthy Controls; ES, Effect Size. η² values of .01, .06, and .14 indicate small, medium, and large effects. Cramér's V values of .10, .30, and .50 indicate small, medium, and large effects. Cohen's d values of .20, .50, and .80 indicate small, medium, and large effects. *p < .05.

Although overall group differences in ADHD prevalence reached statistical significance (χ²(3) = 12.48, p = .006, V = .31), this effect was driven by the absence of ADHD in healthy controls rather than by differential rates among clinical groups, which did not differ significantly from one another (χ²(2) = 1.73, p = .421). Anxiety disorder rates did not differ significantly across groups (χ²(3) = 6.42, p = .093). These patterns suggest that comorbid ADHD and anxiety disorders were distributed comparably across clinical groups and are unlikely to systematically confound between-group differences in the primary outcomes.

Healthy controls were recruited from the same catchment area and screened to confirm the absence of current psychiatric diagnosis and lifetime SUD.

### Measures and clinical assessment

Emotion regulation difficulties were assessed using the Difficulties in Emotion Regulation Scale (DERS), a 36-item self-report instrument measuring non-acceptance, goal-directed behavior difficulties, impulse control difficulties, limited access to regulation strategies, lack of emotional awareness, and lack of emotional clarity. Higher scores indicate greater emotion dysregulation. The Turkish version has demonstrated satisfactory psychometric properties (23); internal consistency in the present sample was α = .81. Impulsivity was measured with the Barratt Impulsiveness Scale-11 (BIS-11), a 30-item self-report instrument assessing attentional, motor, and non-planning impulsivity (24; α = .79 in this sample). Substance use severity was evaluated using the Addiction Profile Index–Adolescent Form (BAPI-E; 25), a 33-item multidimensional measure validated for Turkish adolescents assessing frequency, dependence symptoms, and psychosocial consequences (α = .84 in this sample). The BAPI-E was administered only to participants with active substance use (SUD and BD+SUD groups; n = 64). Depressive and manic symptom severity were assessed using the BDI (26) and YMRS (27), respectively, both validated in Turkish adolescent populations.

Sociodemographic and clinical data were obtained through structured interview and medical record review. Age was recorded in years and sex coded as male/female. Residence was classified as urban or rural according to municipality designation. Family structure was categorized as nuclear versus non-nuclear. Socioeconomic status was indexed by household monthly income (below minimum wage, minimum wage, above minimum wage) and parental educational attainment. Academic functioning was assessed via history of school dropout, grade retention, and current enrollment status. Family psychiatric history among first- and second-degree relatives was documented. Clinical variables including age at BD onset, episode history, hospitalization, suicide attempts, and psychotic features were extracted from records and confirmed during interview. Medication status (mood stabilizers and antipsychotics) was coded dichotomously for sensitivity analyses; as BD groups did not differ significantly on medication variables (all p >.50), medication was not retained in primary models.

To address the potential confounding influence of pharmacological dosage and treatment duration, both variables were additionally recorded and compared between the two BD groups (BD+SUD vs. BD-only). No significant between-group differences were found in mean daily mood stabilizer dose (expressed as lithium-equivalent or chlorpromazine-equivalent units where applicable), mean daily antipsychotic dose, or treatment duration in months (all p >.05). These null findings provided additional justification for excluding medication variables from primary ANCOVA models.

### Statistical analysis

Continuous variables are reported as mean ± standard deviation and categorical variables as frequencies and percentages. Normality was evaluated using the Shapiro–Wilk test. Group differences in categorical variables were examined using chi-square or Fisher’s exact tests, and non-normally distributed continuous variables were analyzed with Kruskal–Wallis tests.

Primary analyses consisted of 2×2 factorial analyses of covariance (ANCOVA) with DERS and BIS-11 total scores as dependent variables, BD and SUD as fixed factors, and age, sex, residence, family structure, and income as covariates due to baseline group differences. Type III sums of squares were used given heterogeneity of variances. Bonferroni correction was applied for *post-hoc* simple effects. Exploratory analyses included correlation analyses, multiple linear regression with BAPI total score as the dependent variable—conducted in the substance-using subsample (SUD-only and BD+SUD groups; n = 64)—and binary logistic regression examining predictors of substance use outcomes. Multicollinearity was evaluated using variance inflation factors (VIF < 3). Effect sizes are reported as partial eta-squared, Cohen’s d, Cramér’s V, and R² where appropriate. Statistical significance was set at α = .05 (two-tailed). Analyses were conducted using R version 4.5.2.

### Ethical considerations

The study was approved by the Ege University Faculty of Medicine Clinical Research Ethics Committee (Application No: 2025-4776). Written informed consent was obtained from all participants and their legal guardians. Data were anonymized and handled in accordance with confidentiality standards. Reporting followed STROBE guidelines.

## Results

### Sociodemographic and clinical characteristics

Groups did not differ significantly in age (SUD: 15.8 ± 1.3; BD+SUD: 14.9 ± 1.6; BD: 15.3 ± 1.7; HC: 15.8 ± 1.1; F = 2.58, p = .056, η² = .06) or sex distribution (χ² = 5.60, p = .133), indicating initial demographic comparability (**Table 1**).

Significant group differences emerged across socioeconomic indicators. Residence (urban vs. rural), family structure, and monthly income differed across groups (all p <.001 to.004). Participants in the SUD and BD+SUD groups were significantly more likely to come from non-nuclear family structures (p = .004) and low-income households (p <.001) compared to the BD and HC groups. Parental education was also significantly lower in SUD-related groups (maternal education: p <.001), suggesting a clustering of socioeconomic disadvantage in substance-related profiles. Because these variables were unevenly distributed across groups, residence, family structure, and income were included as covariates in subsequent ANCOVA models.

Academic and functional indicators further differentiated the groups. School dropout, grade retention, and employment status differed significantly across groups (all p <.001; **Table 1**). Both SUD and BD+SUD groups showed markedly higher school dropout and grade retention rates, consistent with substance-associated academic impairment.

With respect to familial psychiatric burden, the BD+SUD group displayed the most adverse profile. Compared to other groups, this group showed higher rates of paternal psychiatric diagnosis (p = .001), paternal substance use (p = .004), maternal psychiatric disorder (p <.001), and maternal alcohol use (p = .038). This pattern suggests an intergenerational clustering of psychiatric risk in adolescents with comorbid BD and SUD.

### Pharmacological treatment profile

A direct comparison of the two bipolar groups (BD and BD+SUD) revealed that BD onset age was significantly earlier in the comorbid group (13.6 ± 1.6 vs. 14.5 ± 1.7 years; t = −2.48, p = .015, d = 0.62). Critically, the two groups did not differ significantly in type of mood stabilizer (p = .540) or antipsychotic medication (p = .834), or in rates of combination antipsychotic use (BD+SUD: 56.3% vs. BD: 53.1%; p = .812). Mood stabilizer use was high in both groups (BD+SUD: 84.4%; BD: 81.3%), as was antipsychotic use (BD+SUD: 93.8%; BD: 90.6%). The absence of between-group differences in pharmacological treatment type reduces the likelihood that medication status confounded the observed differences in impulsivity and emotion regulation between the two BD groups.

Supplementary analyses further revealed no significant between-group differences in mean daily mood stabilizer dose, antipsychotic dose, or treatment duration (all p >.05), extending the pharmacological equivalence finding beyond medication type to dosage and duration parameters.

### Substance use profiles

When comparing substance use patterns between the SUD-only and BD+SUD groups, the comorbid group demonstrated a markedly more diverse and high-risk use profile (**Table 2**). Nicotine (90.6% vs. 65.6%; p = .016), ecstasy (56.3% vs. 18.8%; p = .003), amphetamine (65.6% vs. 31.3%; p = .008), and heroin (28.1% vs. 0%; p = .001) use were all significantly more prevalent in the BD+SUD group. Benzodiazepine misuse (43.8% vs. 9.4%; p = .002), inhalant use (46.9% vs. 12.5%; p = .003), and opioid use (37.5% vs. 12.5%; p = .021) were also significantly elevated. All significant between-group differences exhibited medium effect sizes (Cramér’s V ≈.29–.40). Cannabis and alcohol use did not differ significantly between groups (both p >.50).

**Table 2:** Substance use type by group (SUD only vs. BD+SUD).

Substance	SUD Only (n=32)	BD+SUD (n=32)	χ²	p	V
Nicotine	21 (65.6%)	29 (90.6%)	5.85	.016*	.30
Ecstasy	6 (18.8%)	18 (56.3%)	9.08	.003*	.38
Amphetamine	10 (31.3%)	21 (65.6%)	7.01	.008*	.33
Heroin	0 (0.0%)	9 (28.1%)	10.47	.001*	.40
Inhalants	4 (12.5%)	15 (46.9%)	9.05	.003*	.38
Benzodiazepines	3 (9.4%)	14 (43.8%)	9.69	.002*	.39
Opioids	4 (12.5%)	12 (37.5%)	5.33	.021*	.29
Cannabis	22 (68.8%)	20 (62.5%)	0.29	.591	.05
Alcohol	24 (75.0%)	22 (68.8%)	0.34	.562	.07

Data are n (%). *p < .05. Cramér's V: .10 = small, .30 = medium, .50 = large.

### Bipolar episode and illness course profile

The two bipolar groups differed notably in their episode profiles (**Table 3**). Mixed-feature episodes were predominant in both groups, but more pronounced in the BD+SUD group (BD+SUD: 62.5%; BD: 34.4%). Depressive episodes as the predominant type were absent in the BD+SUD group but present in 37.5% of the BD-only group. Illness course was broadly similar between groups: 81.3% of BD+SUD participants and 75.0% of BD-only participants achieved full interepisode recovery. Psychotic features were significantly more common in the BD+SUD group (21.9% vs. 3.1%; p = .023). High-lethality suicide attempts were observed exclusively in the BD+SUD group (15.6% vs. 0%; Fisher exact p = .015), though overall rates of any suicide attempt did not reach statistical significance (50.0% vs. 31.2%; p = .120).

**Table 3:** Bipolar episode characteristics and illness course.

Feature	BD+SUD (n=32)	BD Only (n=32)
Predominant episode type		
Manic/Hypomanic	12	9
Mixed Features	20	11
Depressive	0	12
Index episode type		
Manic/Hypomanic	9	4
Mixed Features	20	25
Depressive	3	3
Illness course		
Full interepisode recovery, n (%)	26 (81.3)	24 (75.0)
Partial recovery / persistent impairment, n (%)	6 (18.7)	8 (25.0)

Mixed features defined per DSM-5 criteria.

### Descriptive statistics for clinical scales

Internal consistency coefficients were acceptable to high across scales (DERS: α = .81; BIS-11: α = .79; BAPI: α = .84). All clinical group participants were confirmed to be in a euthymic state at the time of assessment. Both BD groups had mean YMRS scores below the euthymia threshold of 7, and mean BDI scores were below the clinical threshold of 10 across all groups (**Table 4**).

**Table 4:** Descriptive statistics for clinical scales across groups (M ± SD).

Scale	SUD (n=32)	BD+SUD (n=32)	BD (n=32)	HC (n=32)
DERS Total (α = .81)	69.5 ± 20.2	106.0 ± 40.9	48.9 ± 5.0	40.1 ± 2.6
BIS-11 Total (α = .79)	61.9 ± 21.4	87.2 ± 25.9	40.1 ± 6.3	31.2 ± 2.4
BAPI Total (α = .84)	55.2 ± 15.8	84.0 ± 26.0	n/a	n/a
BDI	4.0 ± 2.6	4.6 ± 2.1	6.4 ± 1.4	3.2 ± 2.1
YMRS	3.1 ± 1.2	3.4 ± 2.9	2.3 ± 1.1	1.3 ± 1.1

DERS, Difficulties in Emotion Regulation Scale; BIS-11, Barratt Impulsiveness Scale-11; BAPI, Addiction Profile Index; BDI, Beck Depression Inventory; YMRS, Young Mania Rating Scale. BAPI scores are not applicable for BD and HC groups as these participants had no current substance use. All groups were assessed during euthymia (YMRS ≤ 7; BDI ≤ 10).

DERS total scores were highest in the BD+SUD group (106.0 ± 40.9), followed by SUD (69.5 ± 20.2), BD (48.9 ± 5.0), and HC (40.1 ± 2.6). Similarly, BIS-11 total scores were highest in the BD+SUD group (87.2 ± 25.9), followed by SUD (61.9 ± 21.4), BD (40.1 ± 6.3), and HC (31.2 ± 2.4). This pattern motivated the subsequent ANCOVA interaction analyses. Across the full clinical sample (n = 96), DERS and BIS-11 total scores were moderately and positively correlated (r = .40, p <.001).

### Multivariate analyses: emotion regulation, impulsivity, and clinical severity

To examine the independent and interactive effects of BD status and SUD status on emotion regulation and impulsivity, 2×2 factorial ANCOVAs were conducted with age, sex, family structure, residence, and income entered as covariates. Levene’s test indicated heterogeneity of error variances across groups (all p <.001); accordingly, Type III sums of squares were used. Bonferroni corrections were applied to simple effects comparisons within the ANCOVA models. Correlational and regression analyses were conducted as exploratory and should be interpreted with appropriate caution given the number of tests performed.

### Emotion regulation (DERS)

The ANCOVA model revealed a strong main effect of SUD, F(1,120) = 102.96, p <.001, ηp² = .462. The main effect of BD did not reach significance after covariate adjustment, F(1,120) = 2.95, p = .088, ηp² = .024. A significant BD × SUD interaction was found, F(1,120) = 35.89, p <.001, ηp² = .230.

**Figure 1:**
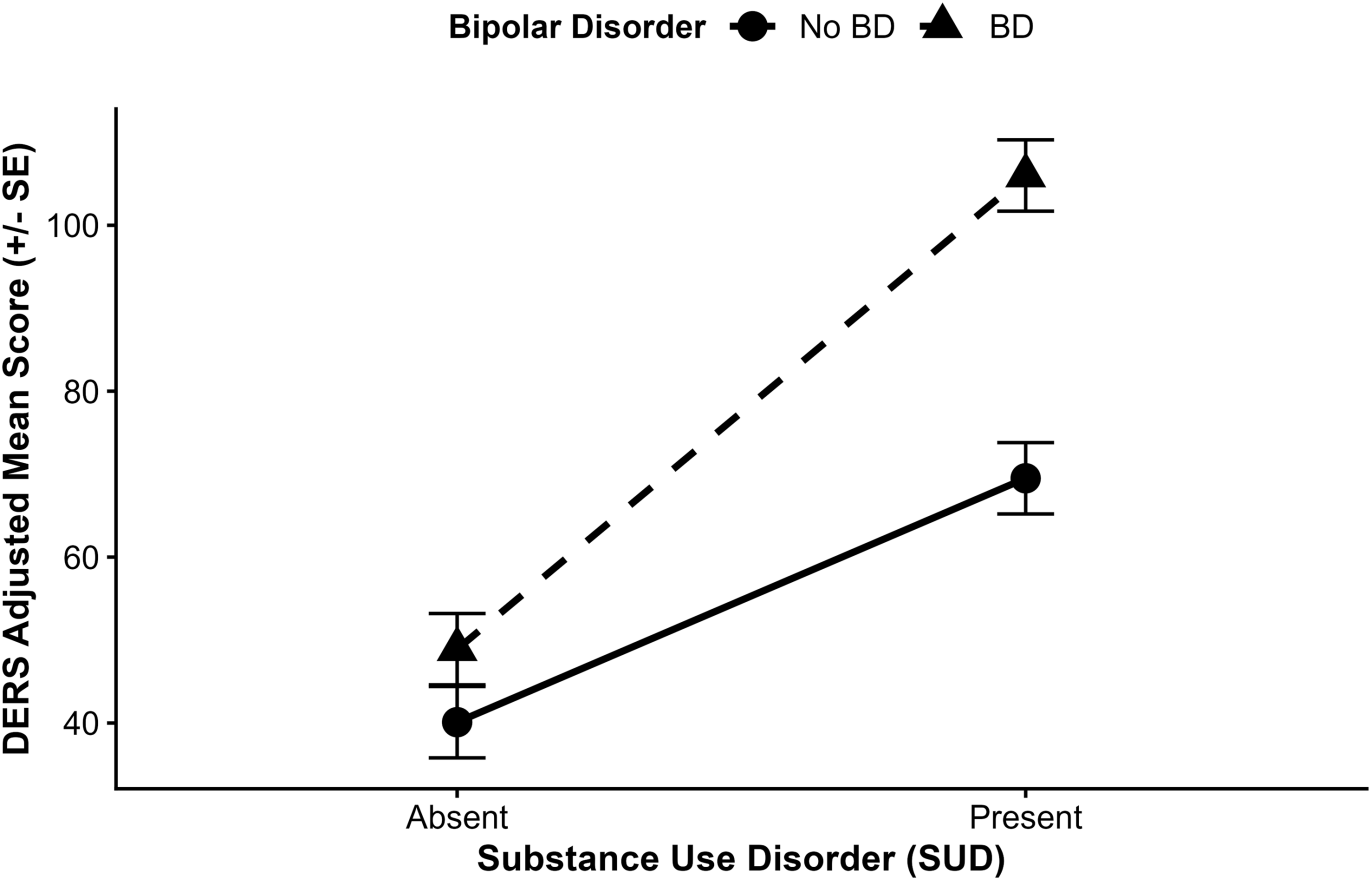
Adjusted mean Difficulties in Emotion Regulation Scale (DERS) total scores by bipolar disorder (BD) and substance use disorder (SUD) status. Means are adjusted for age, sex, residence, family structure, and income; error bars represent standard errors. The BD+SUD group showed the highest emotion dysregulation scores.

Bonferroni-corrected simple effects analysis revealed that within the SUD-absent groups, BD presence was not associated with significantly different DERS scores (p = .088). However, within the SUD-present groups, BD presence was associated with significantly higher DERS scores, t(120) = 6.74, p <.001. The BD+SUD group scored significantly higher than the SUD-only group in the adjusted model (mean difference = 38.90 points) (**Figure 1**).

### Impulsivity (BIS-11)

The ANCOVA model for BIS-11 revealed a significant main effect of SUD, F(1,120) = 42.47, p <.001, ηp² = .261. The main effect of BD was not significant, F(1,120) = 2.96, p = .088, ηp² = .024. The BD × SUD interaction was significant, F(1,120) = 9.51, p = .002, ηp² = .073.

**Figure 2:**
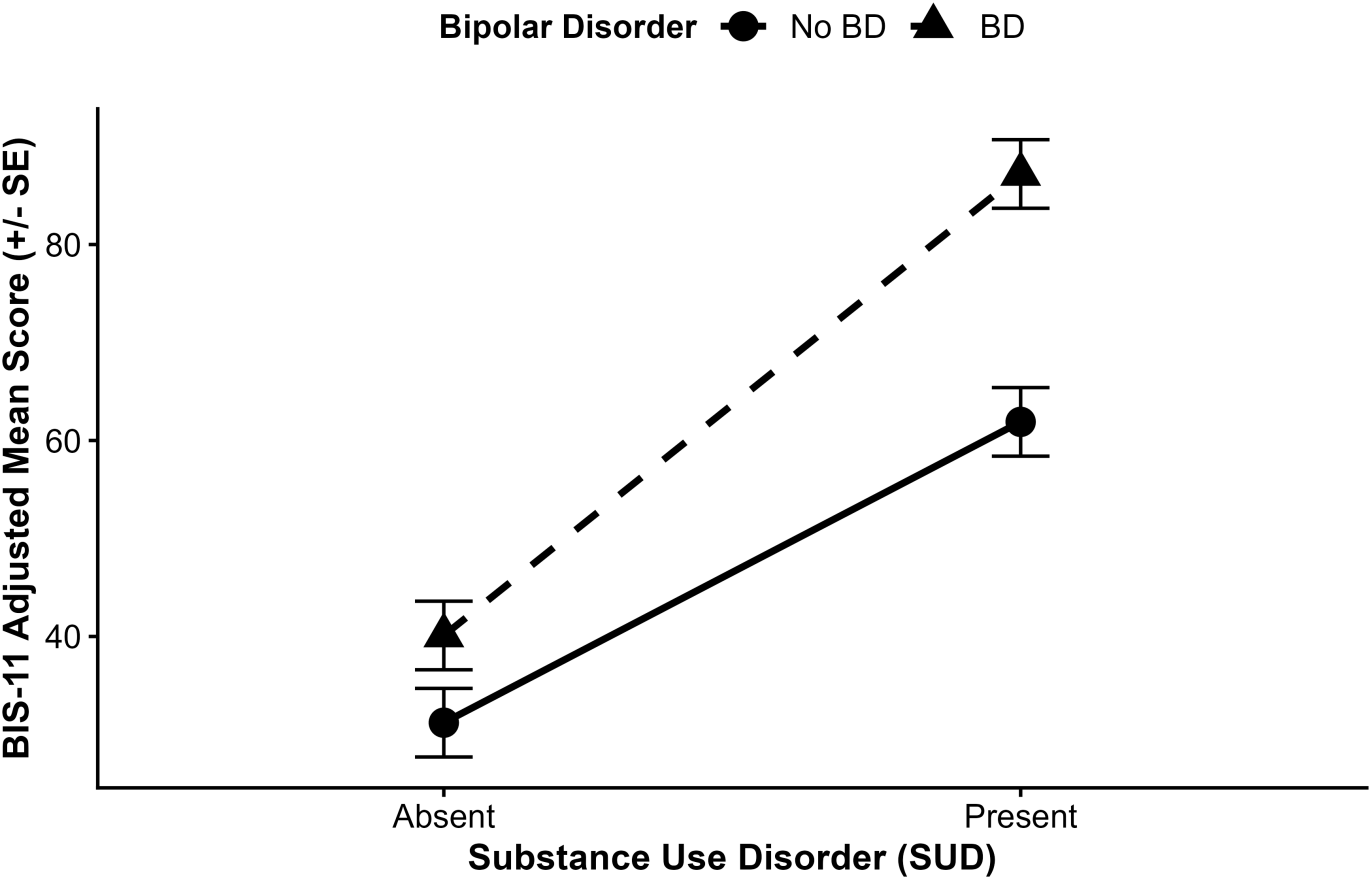
Adjusted mean Barratt Impulsiveness Scale-11 (BIS-11) total scores by bipolar disorder (BD) and substance use disorder (SUD) status. Means are adjusted for age, sex, residence, family structure, and income; error bars represent standard errors. The BD+SUD group showed the highest impulsivity scores.

Simple effects analysis indicated that BD presence was not associated with significantly different BIS-11 scores in the SUD-absent groups (p = .088). In contrast, in the SUD-present groups, BD presence was associated with significantly higher impulsivity scores, t(120) = −6.08, p <.001, with the BD+SUD group scoring significantly higher than the SUD-only group (mean difference = 26.72 points) (**Figure 2**).

### Sensitivity analyses

To assess whether the primary interaction effects remained robust after accounting for residual subthreshold mood symptoms, sensitivity analyses were conducted by additionally entering YMRS and BDI total scores as covariates in the ANCOVA models for both DERS and BIS-11. The BD × SUD interaction remained statistically significant for emotion regulation (p <.001) and impulsivity (p = .002) after inclusion of these mood severity indices, indicating that the observed group differences are not attributable to residual subclinical affective symptoms within the euthymia threshold.

### Regression analyses

To identify predictors of substance use severity (BAPI total), a multiple linear regression was conducted in the substance-using subsample (SUD-only and BD+SUD groups; n = 64), as BAPI-E was administered exclusively to these participants. The model was statistically significant (p <.001) and accounted for 48% of variance in BAPI scores (R² = .48, Adjusted R² = .43, F(8, 55) = 13.24). Multicollinearity was not indicated (all VIF < 3; BIS-11: VIF = 1.19; DERS: VIF = 1.43; BDI: VIF = 1.25). Impulsivity was the strongest positive predictor (B = 0.61, t = 6.53, p <.001), followed by depressive symptoms (B = 0.22, t = 2.38, p = .021). Emotion regulation difficulties showed a significant negative association with BAPI scores (B = −0.22, t = −2.80, p = .007), likely reflecting a statistical suppressor effect: when impulsivity is held constant, the residual variance in DERS scores is inversely related to substance use severity, a pattern commonly observed when correlated predictors share overlapping variance with the outcome.

This suppressor pattern carries a specific clinical implication: it does not suggest that emotion regulation difficulties are protective against substance use severity. Rather, when impulsivity is statistically held constant, the residual variance in DERS scores reflects regulatory difficulties already captured by the impulsivity pathway. Clinically, this underscores that emotion dysregulation and impulsivity should be assessed and treated conjointly rather than as independent predictors, as their shared variance constitutes the most potent driver of substance use severity in this population. Manic symptom severity (YMRS) did not reach significance (p = .192).

Binary logistic regression identified low income as the strongest predictor of clinical group membership (OR = 40.67, 95% CI [6.61–808.71], p = .001). Female sex was also a significant predictor (OR = 5.28, 95% CI [1.45–21.90], p = .015).

## Discussion

The present study examined impulsivity and emotion regulation difficulties across four adolescent groups using a 2×2 factorial ANCOVA design controlling for sociodemographic covariates. The findings largely supported the primary hypotheses regarding impulsivity and revealed an additive pattern in comorbid presentations. Hypothesis 1 was supported: adolescents with BD+SUD demonstrated the highest impulsivity scores (BIS-11), significantly exceeding all other groups after covariate adjustment (mean difference vs. SUD-only = 26.72 points). Contrary to the prediction that SUD-only adolescents would exhibit higher emotion regulation difficulties, the BD+SUD group exhibited the highest DERS total scores, suggesting an additive rather than dissociative effect of co-occurring diagnoses on emotion dysregulation. Hypothesis 2 was supported, as both clinical groups scored significantly higher than healthy controls on primary outcomes. Exploratory regression analyses further indicated that impulsivity, emotion regulation difficulties, and depressive symptoms independently predicted substance use severity (R² = .48), while low income emerged as the strongest predictor of clinical group membership (OR = 40.67).

Together, these findings suggest that comorbid BD+SUD in adolescence is characterized by a compound profile of markedly elevated impulsivity and emotion dysregulation, alongside severe and heterogeneous substance use, earlier BD onset, and mixed-feature predominance, consistent with an additive burden model.

### Additive emotion dysregulation in comorbid BD+SUD

The finding that BD+SUD adolescents reported the greatest emotion regulation difficulties—exceeding even the SUD-only group by a clinically meaningful margin (mean difference = 38.90 points)—is consistent with an additive model of comorbidity (28). Rather than emotion dysregulation being diagnosis-specific, this pattern suggests that the co-occurrence of BD and SUD compounds regulatory deficits beyond the burden conferred by either disorder alone.

Several mechanisms may account for this additive pattern. Within a self-medication framework (29), the combination of mood instability inherent to BD and the neuroadaptive effects of chronic substance use may jointly impair emotion regulation capacity (30). Adolescents with BD+SUD face both endogenous affective dysregulation and substance-induced disruptions to prefrontal–limbic circuitry, potentially creating a compounding cycle of regulatory impairment.

The predominance of mixed-feature episodes in the BD+SUD group (62.5%) may further contribute to elevated DERS scores. Mixed states involve simultaneous activation and dysphoria (31), a combination that may be particularly difficult to regulate and that is associated with heightened subjective distress. Additionally, the earlier illness onset in the BD+SUD group (mean = 13.6 years; cf. 32) implies a longer duration of mood instability during a neurodevelopmentally sensitive period, potentially disrupting the consolidation of adaptive regulatory strategies. A summary of the multivariate analyses is presented in **Table 5**.

**Table 5:** Summary of multivariate analyses.

Analysis	Outcome	Effect / Predictor	Statistic	p / ES
ANCOVA				
ANCOVA	DERS	BD (Main Effect)	F(1,120) = 2.95	.088 / ηp² = .024
		SUD (Main Effect)	F(1,120) = 102.96	< .001* / ηp² = .462
		BD × SUD	F(1,120) = 35.89	< .001* / ηp² = .230
ANCOVA	BIS-11	BD (Main Effect)	F(1,120) = 2.96	.088 / ηp² = .024
		SUD (Main Effect)	F(1,120) = 42.47	< .001* / ηp² = .261
		BD × SUD	F(1,120) = 9.51	.002* / ηp² = .073
Multiple linear regression (outcome: BAPI)				
OLS	BAPI	BIS-11 Total	B = 0.61, t = 6.53	< .001* / R² = .48
		BDI Total	B = 0.22, t = 2.38	.021*
		DERS Total	B = −0.22, t = −2.80	.007*
		YMRS Total	B = 0.21, t = 1.32	.192
Binary logistic regression (outcome: clinical group)				
Logistic	Group	Income (Low vs. High)	OR = 40.67	.001*
		Sex (Female vs. Male)	OR = 5.28	.015*

ANCOVA simple effects were Bonferroni-corrected. Logistic and correlational analyses were exploratory. *p < .05. ηp², partial eta-squared. DERS, Difficulties in Emotion Regulation Scale; BIS-11, Barratt Impulsiveness Scale-11; BAPI, Addiction Profile Index; BDI, Beck Depression Inventory; YMRS, Young Mania Rating Scale; OR, Odds Ratio.

Notably, despite presenting the highest DERS scores, the BD+SUD group was also the most impulsive (BIS-11), indicating that both regulatory domains—emotion regulation and inhibitory control—are concurrently impaired. This dual-deficit profile is consistent with theoretical models proposing that impulsivity and emotion dysregulation share common neural substrates in prefrontal–limbic networks (18, 19), and that disruption of these systems in BD+SUD produces a compound phenotype exceeding the sum of its individual components.

Importantly, the cross-sectional design precludes differentiation between trait-level dysregulation and state-dependent reporting effects. Multi-method longitudinal studies incorporating behavioral tasks, physiological indices, and ecological momentary assessment are needed to clarify whether emotion regulation deficits represent premorbid vulnerability, consequence of substance exposure, or state-related distortion.

### Impulsivity as a core amplifier of comorbidity

Impulsivity emerged as the most robust differentiating feature of BD+SUD. The 26.72-point elevation relative to SUD-only is clinically substantial and consistent with models positing additive or synergistic disinhibition in comorbid presentations (33, 34). Impulsivity in BD has been conceptualized as a trait vulnerability persisting across mood states, including euthymia (21, 35). Comorbid SUD may compound this vulnerability through dopaminergic sensitization, progressive disruption of prefrontal inhibitory control, and reward-circuit dysregulation (19, 20).

Adolescence is characterized by asynchronous maturation of limbic reward systems relative to prefrontal regulatory networks (6, 36). Early-onset BD (mean onset 13.6 years in BD+SUD) may further disrupt this developmental trajectory. The predominance of mixed-feature episodes—combining activation with dysphoria—creates a behavioral context conducive to risk-taking and substance misuse. Together, these findings are consistent with a developmental “double-hit” model in which trait impulsivity and affective activation converge to amplify substance-related risk (7, 36).

Given that impulsivity independently predicted substance use severity (B = 0.61, p <.001), its assessment should be routine in adolescents with BD, including during euthymic periods (37).

### Substance use severity and heterogeneity

The BD+SUD group demonstrated markedly greater substance use severity and heterogeneity, including exclusive heroin use (28.1%) and significantly higher rates of amphetamines, ecstasy, benzodiazepine misuse, inhalants, and opioids. These findings align with adult literature linking BD to polydrug use and preference for mood-activating or destabilizing substances (38, 39).

However, caution is warranted. Exclusive heroin use may reflect referral patterns, regional substance availability, or severity-based treatment pathways rather than diagnosis-specific vulnerability. Replication in multi-site samples is necessary before inferring substance preference mechanisms.

Notably, cannabis and alcohol use did not differ between groups, suggesting these substances may be influenced by shared adolescent risk factors rather than diagnosis-specific mechanisms (38).

Beyond descriptive characterization, the elevated rates of stimulant use (amphetamine: 65.6%; ecstasy: 56.3%) and greater polysubstance use in the BD+SUD group may constitute a partial mechanistic explanation for the additive impulsivity and emotion dysregulation profile observed. Stimulant substances exert acute and chronic effects on dopaminergic and noradrenergic circuitry, producing neuroadaptive changes in prefrontal inhibitory control and reward sensitivity that parallel and potentially compound the neurodevelopmental vulnerabilities inherent to early-onset BD (20, 36). Repeated stimulant exposure during a critical period of prefrontal maturation may therefore not merely co-occur with elevated impulsivity, but actively amplify it through progressive catecholaminergic dysregulation. This interpretation is consistent with the double-hit model discussed above and suggests that the substance use profile of BD+SUD adolescents may function as a proximal amplifier of impulsivity—not simply its consequence.

The presence of high-lethality suicide attempts exclusively in BD+SUD (15.6%) underscores the clinical gravity of this constellation (40). The convergence of impulsivity, polydrug use, and mixed episodes may represent a particularly high-risk profile for severe self-harm.

However, the exclusive occurrence of high-lethality attempts in the BD+SUD group should be interpreted in light of the study’s sampling context. Participants were recruited from a tertiary psychiatric and addiction medicine service, which disproportionately concentrates patients with severe, treatment-refractory, or multiply comorbid presentations. It is therefore possible that the observed pattern reflects a severity-based referral gradient rather than a diagnostic-category effect that would generalize to community or primary care settings. Tertiary samples are known to overrepresent patients whose clinical trajectory has already been shaped by hospitalizations, failed outpatient treatment, and crisis-level presentations—all factors that independently elevate suicide attempt lethality (41). Replication in multi-site samples spanning primary, secondary, and tertiary care levels is necessary to determine whether the exclusive concentration of high-lethality attempts in the BD+SUD group is a robust epidemiological finding or a sampling artifact. In the interim, clinicians working in tertiary addiction and mood disorder services should treat BD+SUD comorbidity as a sentinel indicator warranting systematic lethality assessment and lethal-means counseling, irrespective of the underlying causal mechanism.

### Pharmacological equivalence and internal validity

A key methodological strength is the absence of significant differences in pharmacological treatment between the two BD groups. Rates of mood stabilizers, antipsychotics, and combination therapy were statistically equivalent, reducing the likelihood that between-group differences reflect medication confounding. Supplementary analyses additionally confirmed no significant differences in daily medication dose or treatment duration between the two BD groups (all p >.05), extending the equivalence argument beyond treatment type.

Nonetheless, formal adherence monitoring was not conducted, and residual confounding cannot be excluded. Even so, the persistence of large impulsivity differences across pharmacologically comparable groups strengthens the interpretation that comorbidity confers additive neurobehavioral burden beyond treatment effects.

### Developmental and phenotypic considerations

The BD+SUD group was characterized by earlier BD onset, mixed-feature predominance, psychotic features (21.9%), and high-lethality suicide attempts. The near absence of depressive-predominant episodes in this group suggests a profile driven more by activation than inhibition. Rather than representing BD with an incidental comorbidity, this pattern may reflect a high-risk developmental subtype within the BD spectrum characterized by early onset, mixed predominance, and additive impulsivity burden (42). Longitudinal studies are required to determine whether this represents a stable phenotype or a severe developmental stage within the illness trajectory.

### Socioeconomic adversity and contextual amplification

Low income emerged as the strongest predictor of clinical group membership (OR = 40.67), though the wide confidence interval indicates model instability and necessitates cautious interpretation. As participants were recruited through clinical services, socioeconomic differences may partly reflect differential healthcare access and referral pathways rather than population-level prevalence differences.

Nevertheless, clustering of adversity indicators—lower parental education, non-nuclear family structures, familial psychiatric burden, and school dropout (43)—supports an accumulation-of-risk framework (44, 45). Gene–environment interaction models suggest that early-onset BD in high-adversity contexts may confer particularly elevated vulnerability to substance misuse (42). These findings underscore the importance of ecological screening and family-level intervention.

### Clinical implications

Several clinical implications emerge from these findings:

1. Impulsivity-focused assessment and intervention should be prioritized in BD+SUD adolescents. Adapted DBT and inhibitory-control–targeted approaches may be particularly beneficial (28).
2. Emotion regulation interventions may require emphasis across both BD+SUD and SUD-only adolescents, given their elevated DERS scores relative to healthy controls.
3. Integrated dual-diagnosis treatment models are indicated, as sequential or parallel approaches may inadequately address the additive burden observed in BD+SUD (46, 47).
4. Suicide risk assessment must include method lethality, and lethal-means counseling should be systematically incorporated into care for BD+SUD adolescents.

### Limitations

The cross-sectional design precludes causal inference. The single-center clinical sample limits generalizability and introduces potential referral bias.

This concern is particularly relevant to the finding of exclusive high-lethality suicide attempts in the BD+SUD group; the severity-based referral patterns inherent to tertiary psychiatric services may amplify the apparent magnitude of this association relative to what would be observed in population-representative or community samples.

All primary outcomes relied on self-report, raising the possibility of mood-state–dependent reporting effects. Although euthymia was verified, residual subclinical symptoms cannot be excluded.

A related concern pertains to the potential influence of subclinical mood symptoms on self-report accuracy. Although euthymia was operationally defined and verified via YMRS ≤ 7 and BDI ≤ 10, these thresholds do not preclude residual subclinical symptoms within the accepted range. Minor fluctuations in mood state—even below clinical thresholds—may systematically influence subjective perception of emotional clarity and impulse control, thereby introducing measurement variance into DERS and BIS-11 scores that cannot be disentangled from stable trait-level differences in a cross-sectional design. Future studies should consider repeated assessments across multiple euthymic time points or include physiological indices to corroborate self-report findings.

The sample size (n = 32 per group) limits power for detecting small interaction effects.

The logistic regression identifying low income as the strongest predictor of clinical group membership (OR = 40.67, 95% CI [6.61–808.71]) should be interpreted with particular caution. The extremely wide confidence interval is consistent with model instability, likely attributable to the small cell frequencies produced by three-category income classification across four groups (n = 32 per group). This finding may reflect sparse data rather than a stable population-level effect, and should not be interpreted as a reliable point estimate. Replication in larger, population-representative samples using continuous or finer-grained socioeconomic indices is required before any inferential weight can be placed on this association.

The distribution of comorbid diagnoses including ADHD and anxiety disorders across groups is reported in **Table 1**. Rates were comparable across clinical groups, which reduces—though does not eliminate—their potential confounding influence on impulsivity and emotion regulation outcomes.

## Conclusion

Using a factorial design, this study disentangled additive and interactive effects of BD and SUD in adolescence. The findings indicate that comorbid BD+SUD is characterized by markedly elevated impulsivity and emotion dysregulation, severe and heterogeneous substance use, earlier illness onset, and mixed-feature predominance, consistent with an additive comorbidity model.

Both impulsivity and emotion dysregulation appear to function as transdiagnostic dimensions that are additively amplified in comorbid BD+SUD. Socioeconomic adversity and intergenerational psychiatric burden further contextualize vulnerability.

These results support the clinical utility of impulsivity-focused, integrated dual-diagnosis interventions for adolescents with BD+SUD and highlight the need for longitudinal, multi-method research to clarify developmental trajectories and mechanistic pathways underlying this high-risk phenotype.

Future research should prioritize multi-method assessment designs that integrate objective neurocognitive paradigms—such as the Go/No-Go task and stop-signal task, which provide behavioral indices of response inhibition independent of self-report bias (19)—alongside ecological momentary assessment (EMA) methodologies to capture real-time fluctuations in impulsivity and emotional reactivity in naturalistic contexts (48). Such approaches would help clarify whether the elevated impulsivity and emotion dysregulation observed in the BD+SUD group represent stable trait-level deficits, state-dependent reporting effects, or dynamic regulatory failures that emerge in response to daily stressors—a distinction that cross-sectional self-report designs cannot resolve. The present findings should also be interpreted in light of the diagnostic complexity of bipolar disorder (49), the influence of sociodemographic and residential context on psychiatric risk (50), and heterogeneous developmental links among adolescent substance use, depressive symptoms, externalizing psychopathology, and impulsivity (51–53). SUD comorbidity may further worsen cognitive and functional outcomes in bipolar disorder (54), while nonmedical prescription drug use, treatment dropout, and cannabis-associated earlier bipolar onset remain clinically relevant concerns for adolescent dual-diagnosis care (55–57).

## Data Availability

The raw data supporting the conclusions of this article will be made available by the authors, without undue reservation.
